# Overlooked by nurses: A scoping review on health stressors, problems and coping of migrant domestic workers

**DOI:** 10.1002/nop2.1391

**Published:** 2022-09-30

**Authors:** Ken Hok Man Ho, Ingrid M. Wilson, Janet Yuen‐Ha Wong, Lisa McKenna, Sonia Reisenhofer, Ferry Efendi, Graeme D. Smith

**Affiliations:** ^1^ Nethersole School of Nursing, Faculty of Medicine The Chinese University of Hong Kong Hong Kong Hong Kong SAR; ^2^ Health and Social Services Cluster Singapore Institute of Technology Singapore Singapore; ^3^ School of Nursing & Health Studies Hong Kong Metropolitan University Hong Kong Hong Kong SAR; ^4^ School of Nursing and Midwifery La Trobe University Melbourne Victoria Australia; ^5^ Community Wellbeing & Partnerships Bairnsdale Regional Health Service Bairnsdale Victoria Australia; ^6^ Faculty of Nursing Universitas Airlangga Surabaya Indonesia; ^7^ School of Health Sciences Caritas Institute of Higher Education Hong Kong Hong Kong SAR

**Keywords:** community nursing, marginalization, mental health, migrant domestic workers, peer support, physical health, scoping review

## Abstract

**Aim:**

The first scoping review is to map and synthesize the stressors, problems and coping strategies surrounding the health issues of migrant domestic workers.

**Design:**

Scoping review using Arksey and O'Malley's five‐stage framework.

**Methods:**

Ten electronic databases were systematically searched by keywords for literature published between January 1995 and December 2019. Data were extracted into tables and collated and summarized into themes for presentation.

**Results:**

Twenty‐seven reports were included in the final review. Analysis revealed that stressors to health included abuse, poor health service accessibility, ongoing financial hardship despite demanding working conditions and social isolation. Physical and mental health problems were identified for which migrant domestic workers largely depended on social networks and religion to cope with stressors and health problems. Training para‐professional peer leaders of migrant domestic workers by community nurses and including them in interprofessional teams is a possible way for nurses to promote their health and well‐being.

## 1 INTRODUCTION

Since Hoschschild introduced the concept of the global care chain as “a series of personal links between people across globe based on the paid or unpaid work of caring” (Hochschild, 2000, p. 131), migrant domestic workers (MDWs) have become a major international target population for issues of human rights. MDWs are an emerging group of caregivers in high‐income countries and a vulnerable group of ethnic minorities facing extreme health inequity (Gallotti, 2015; Ho & Smith, 2020). An MDW can be defined as a full‐time worker who is tied to an employer either through a regulated work permit or an underground contract that allows him/her to work for a single household (Basnyat & Chang, 2017). There are an estimated 11.5 million MDWs in the world, of whom 75% are female (Gallotti, 2015). Studies of MDWs have uncovered systemic inequity based on gender, economic class, ethnic group and legal status (Palenga‐Möllenbeck, 2013), exacerbating the health inequalities they face (Chung & Mak, 2020). Examples of health inequalities of MDWs included poor accessibility to health and social care services and poor mental health (e.g. loneliness, depression, anxiety) (Ho et al., 2022). Tackling the health inequalities and inequity of MDWs requires collaborations between health and social care professionals (e.g. nurses and social workers) at the research, practice and policy level (Cheung et al., 2019; Ho & Smith, 2020). Therefore, we reviewed stressors, problems and coping surrounding health issues of MDWs to propose possible strategies to intervene.

Generally, the work of MDWs includes household chores and caring (Ho et al., 2019). People from Poland have been employed as domestic workers in Germany to take care of older adults (Palenga‐Möllenbeck, 2013). In Scandinavia, Sweden provides tax reductions for older adults to pay for “top‐up” care services from migrant workers (Gavanas, 2013). Filipina domestic workers have been employed to care for older adults in Cyprus (Panayiotopoulos, 2005) and Israel (Ayalon & Roziner, 2016). Approximately 14% (*n* = 183,000) of Hong Kong older adults are reportedly cared for by MDWs (Ho et al., 2021). The number of MDWs hired to specifically care for older adults was projected to increase from 198,000 in 2011 to 300,000 by 2030 in Singapore (National Population and Talent Division, 2012). Around 12.8% of frail older adults in Taiwan were under the care of migrant domestic workers (Chou et al., 2015) and 17.4% of Israeli older adults received government subsidies to hire MDWs (Ayalon & Roziner, 2016).

The role of MDWs has a unique nature, as both employees and caregivers of their employer or the family members of employers, requiring MDWs to employ heavy emotional labour in their relationships with care recipients (Ho et al., 2019). As such, the mental health of MDWs is a major issue. Half of the MDWs in China reportedly found it difficult to manage negative emotions (Wang & Wu, 2017). MDWs were reportedly highly vulnerable to abuses by employers (Ho et al., 2022). In fact, abusive behaviours by employers, such as physical and verbal abuse, sexual harassment and economic exploitation were identified as common stressors that trigger negative emotions among MDWs (Wang & Wu, 2017). Due to their live‐in working arrangements, MDWs provide round‐the‐clock care to recipients, potentially having an impact on their physical health status. For example, 52% of MDWs complained of a lack of sleep and 73% reported not having regular mealtimes, due to the constant demands of their caregiving work (Mission for Migrant Workers, 2018).

Both social sciences (e.g. Ayalon & Roziner, 2016) and medicine (e.g. Cheung et al., 2019) have independently investigated the health issues of MDWs. While nurses have an important responsibility to promote and protect the mental and physical health of vulnerable groups (e.g. ethnic minorities and caregivers) (Douglas et al., 2014), there are no examples of input from nurses. It has also been highlighted that healthcare professionals (e.g. nurses) and current healthcare services and policies were unable to meet the needs of MDWs (Cheung et al., 2019; Ho & Smith, 2020). Given the increasing global trend of hiring MDWs, coupled with a lack of systematic information on the health issues of MDWs (Gallotti, 2015), this is the first scoping review on health stressors and health problems affecting MDWs worldwide in order to suggest potential interventions for nurses to mitigate the health inequity faced by MDWs.

## 2 METHODS

We employed Arksey and O'Malley's (2005) five‐stage framework and followed the advice of Levac et al. (2010) to synthesize and analyse a wide range of literature. Arksey and O'Malley (2005) proposed five stages to conduct a scoping review: (1) identifying the research question; (2) identifying relevant studies; (3) selecting studies; (4) charting the data; and (5) collating, summarizing and reporting the results. Underpinned by the framework (Arksey & O'Malley, 2005), this study is reported according to PRISMA reporting (Page et al., 2021). Steps one to five of this five‐stage framework (Arksey & O'Malley, 2005) are described below.

### 2.1 Identifying the research question

This scoping review aimed to synthesize the stressors, problems and coping strategies surrounding the health issues of migrant domestic workers internationally. The research questions addressed by this review were:

- What is known about the stressors impacting the health of MDWs?
- What are the health problems faced by MDWs?
- What are the coping strategies employed by MDWs in response to stressors and health problems?

### 2.2 Identifying relevant studies

Relevant studies were identified principally by systematically searching electronic databases including Scopus, Web of Science, CINAHL, Medline, PsychInfo, Proquest, PubMed, Garuda (Indonesian Publication Database), Business Source Complete and China Journals Full‐text Database with the publications from January 1995 to December 2019. Various combinations of the medical subject headings (MeSH) with Boolean operators AND and OR were used: “domestic helper,” “domestic worker,” “migrant worker,” “foreign worker,” “domestic employee,” “health,” “well‐being,” “psych*,” “mental,” “emotional,” “physical” and “work life.” A second search was also conducted by reviewing bibliographic references of relevant and included studies. Due to the cultural diversity in the team, we were able to extend our search to literature in English, Chinese and Indonesian languages. An example of search is provided in Appendix 1.

### 2.3 Study selection

**FIGURE 1:**
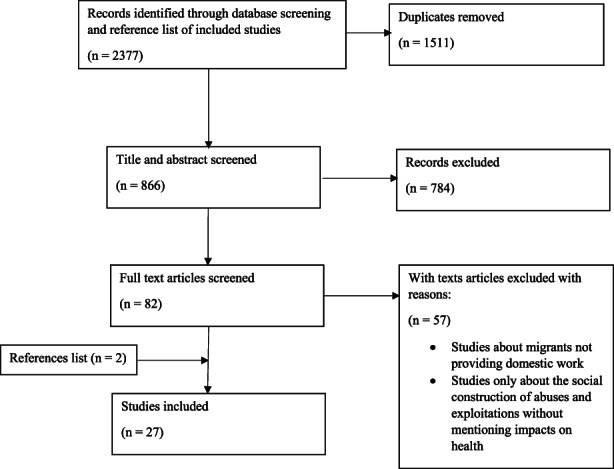
PRISMA flow chart

In total, 2,377 references were obtained for literature in English, Chinese or Indonesian, of which 1,511 were eliminated as duplicates. A study team of seven members screened the titles and abstracts of the remaining 866 articles using the Covidence software platform. Each article was screened independently by two team members with disputes resolved by a third team member. After reviewing titles and abstracts, 82 full‐text relevant articles were retrieved and screened again. Articles were screened based on the following inclusion criteria: (1) only international peer‐reviewed studies and original articles; and (2) research design was quantitative, qualitative or mixed methods studies. Studies were excluded if they: (1) only mentioned social construction of exploitation or abuse on MDWs, (2) involved participants who were migrant workers but not engaged in domestic work or (3) not focussed on the health and well‐being of domestic migrant workers. We also excluded commentaries, discussion papers, editorials, literature reviews and book chapters (Cheung et al., 2021). Monthly meetings were carried out to clarify the study selection eligibility criteria. No Chinese or Indonesian literature met the inclusion criteria. In total, 25 articles were included, and an additional two articles were retrieved by searching on Google. Therefore, 27 articles were included in the final scoping review. The search process is provided in Figure 1.

### 2.4 Charting the data

Characteristics of included studies (Table 1) were extracted into a Microsoft Excel table including data on author, year, study design, research objectives, number of participants, study location and worker country of origin. In accordance with the inductive nature of scoping review (Arksey & O'Malley, 2005), no studies were excluded based on quality. Another table (Table 2) was further developed to extract specific data on stressors on health, health problems and coping strategies. All data charting was conducted independently by two reviewers and results were cross‐checked by other team members.

**TABLE 1:** Characteristics of included literature

Authors	Study design	Research objectives	No. of sample/participants	Study conducting countries	Origin countries of MDWs
Ahonen et al., 2010	Qualitative	To examine the environmental, ergonomic and psychosocial hazards and health effects identified by immigrant women working in household service in five Spanish cities	Forty‐six immigrant women in household services	Spain	Colombia, Morocco, Senegal, Romania, Equador
Anbesse et al., 2009	Qualitative	To explore the experience of female Ethiopian domestic workers employed in Middle Eastern countries and illuminate potential threats to mental health	Nineteen female domestic workers (two groups with severe mental illness and one mentally well group)	Middle East (Lebanon, Saudi Arabia, Abu Dhabi, Yemen)	Ethiopia
Anjara et al., 2017	Quantitative	To investigate factors impacting on female domestic workers' health and quality of life	One hundred eighty‐second female migrant domestic workers	Singapore	Philippines, Indonesia, Myanmar/Sri Lanka
Bagley et al., 1995	Quantitative	To investigate stress factors, satisfaction and mental health adjustment of female domestic helpers	Six hundred female domestic helpers	Hong Kong	Philippines
Carlos & Wilson, 2018	Qualitative	To examine perceived changes in health and barriers to accessing healthcare services	Twenty‐one temporary female live‐in caregivers	Canada	Philippines
Cheung et al., 2019	Quantitative	To explore the experience of physical and verbal abuse and reporting behaviours, and associations between abuse experience and depression‐level	One hundred and five female foreign domestic helpers	Hong Kong	Philippines
Fernandez, 2018	Qualitative	To investigate healthcare needs, access to healthcare and healthcare strategies of migrant domestic workers	Thirty five female domestic workers and 17 key informants	Lebanon	Ethiopia
Gao et al., 2014	Quantitative	To explore the social determinants of oral health of foreign domestic workers	One hundred twenty‐two female domestic helpers	Hong Kong	Indonesia
Garabiles et al., 2019	Quantitative	To explore comorbidity between anxiety and depression among migrant domestic workers	Three hundred fifty‐five female domestic workers	Macao, China	Philippines
Green & Ayalon, 2016	Quantitative	To explore the help‐seeking behaviour of migrant home care workers exposed to workplace abuse	Eighty‐five home care workers (86% female)	Israel	Philippines
Green & Ayalon, 2018	Quantitative	To assess the working conditions and prevalence of abuse and exploitation of live‐in and live‐out migrant home care workers	Three hundred thirty‐eight migrant live‐in home care workers (84% female) and 185 local live‐out workers (92% female)	Israel	Russia (live‐out workers) Not stated for live‐in workers
Hall, Garabiles, & Latkin, 2019	Qualitative	To identify key health issues faced by migrant domestic workers and the social determinants	Twenty‐two female domestic workers; seven key informants	China	Philippines
Hall, Pangan, et al., 2019	Quantitative	To investigate the relationship between discrimination and anxiety and depressive symptoms, and social capital as a moderator	One hundred thirty‐one female domestic workers	Macao, China	Philippines
Heng et al., 2019	Qualitative	To explore caregiving experiences and coping strategies of female domestic workers caring for older people in Singapore	Eleven female domestic workers	Singapore	Indonesia, Philippines and Burma
Hill et al., 2019	Mixed methods	To examine the occupational health and safety experience of migrant live‐in carers	One hundred twelve female domestic workers (eight interviewed)	Canada	Philippines
Kantaris et al., 2014	Quantitative	To investigate the conditions of access and utilization of health services by domestic helpers	Six hundred twenty‐five domestic helpers (98.9% female)	Cyprus	Philippines, Vietnam, Sri Lank, India and Other
Lo et al., 2019	Quantitative	To explore stress levels, social support requirements and perceived quality of life among foreign care workers in home care settings	One hundred fifty‐seven foreign care workers (99.3% female)	Taiwan	Indonesia
Mendoza et al., 2017	Quantitative	To examine the role pf social network support in buffering the impact of postmigration stress on mental health symptoms among female domestic workers.	Two hundred sixty‐one female domestic workers	Macau, China	Philippines
Simkhada et al., 2018	Quantitative	To explore the health problems of Nepalese female migrant workers working in the Middle East and Malaysia	One thousand and ten female migrant workers	Middle East, Kuwait, Saudia Arabia, Malaysia	Nepal
Toyota, 2006	Qualitative	To explore the health and welfare concerns of cross‐border domestic maids in Thailand	Three female domestic workers	Thailand	Burma
Vahabi & Wong, 2017	Mixed methods	To explore work‐related experiences and mental health of female domestic workers	Thirty female domestic workers	Canada	Philippines
Van Bortel et al., 2019	Qualitative	To explore the perceived stressors and coping mechanisms of female migrant domestic workers	One hundred eighty‐second female domestic workers	Singapore	Philippines, Indonesia, Myanmar, Sri Lanka.
van der Ham et al., 2014	Mixed methods	To explore factors that contribute to resilience in female domestic workers and the relationship between stress and well‐being	Five hundred female domestic workers	Philippines	Philippines
van der Ham et al., 2015	Mixed methods	To assess the stress and coping of female migrant domestic workers from the Philippines in different phases of the migration process	Five hundred female domestic workers	Philippines	Philippines
Wong et al., 2020	Quantitative	To assess the acceptability and effectiveness of a 4‐week cognitive behavioural therapy (CBT)‐based para‐professional training programme for Filipina foreign domestic workers	Forty female Filipina domestic workers	Singapore	Philippines
Yi et al., 2019	Quantitative	To investigate the prevalence of gambling disorders in Filipino female domestic workers in Macao	One thousand one hundred ninety‐four female domestic workers	Macao, China	Philippines
Zahreddine et al., 2014	Mixed methods	To assess psychiatric morbidity of female domestic workers	Thirty‐three female domestic workers and 14 Lebanese females (control)	Lebanon	Ethiopia, Bangladesh and Philippines

**TABLE 2:** Data charting on stressors, health problems and coping strategies

Authors	Stressors	Health problems	Coping strategies
Ahonen et al., 2010	*Work environment*: Demanding work and emotional toll from caring for older adults *Social isolation*: Long working hours and lack of interaction with co‐workers contribute to social isolation. Separation from family reinforces the sense of otherness in the host country	*Physical health*: Poor sleep and fatigue related to mental issues *Mental health*: Anxiety and depression	NA
Anbesse et al., 2009	*Financial hardship*: MDW must endure demanding work conditions as unable to quit domestic work due to financial hardship *Social isolation*: Major stressor	Progressive health declines postmigration *Mental health*: Loneliness and homesickness	*Social support*: Establishing social networks and contact with fellow workers and engaging in religious worship together, though social contacts may be prohibited by employers
Anjara et al., 2017	*Social isolation*: Major stressor	*Mental health*: Stress	*Social support*: Higher quality of life for more socially connected MDWs
Bagley et al., 1995	NA	*Mental health*: Stress; anxiety and moderate to severe depression	NA
Carlos & Wilson, 2018	*Access to health services*: Lack of health insurance coverage—low levels of knowledge of MDW entitlement to compulsory health insurance from employers *Social isolation*: Separation from family contributes to social isolation.	*Physical health*: Cardiovascular diseases and poor sleep postmigration *Mental health*: Stress, anxiety and depression	NA
Cheung et al., 2019	*Work‐related abuse*: Physical and verbal abuse	*Mental health*: Moderate to severe depression	NA
Fernandez, 2018	*Access to health services*: Poor access to healthcare arising from structural exclusion from the healthcare system	*Physical health*: Cardiovascular and respiratory diseases and chronic pain postmigration. Reproductive health concerns due to lack of access to health services.	NA
Gao et al., 2014		*Physical health*: Poorer oral health postmigration linked to psychosocial determinants	NA
Garabiles et al., 2019		*Mental health*: Increased anxiety, depression, somatization and post‐traumatic stress disorders associated with postmigration stress	NA
Green & Ayalon, 2016	*Work‐related abuse*: Sexual abuse, physical abuse, emotional abuse and exploitation	NA	*Social support*: Low reporting rate of abuse to formal or informal supports
Green & Ayalon, 2018	*Work‐related abuse*: Migrant domestic workers more vulnerable to abuse than local care workers. Abuse of workers' rights (e.g. lack of written contracts, vacation and sick leave, financial compensation and time off)	NA	NA
Hall, Garabiles, & Latkin, 2019	NA	*Physical health*: Cardiovascular diseases, chronic pain, diabetes postmigration *Mental health*: Anxiety and depression, moderate to severe depression. Mental health issues coupled with poor social support contributed to addictive gambling	*Social support*: Peer sharing of same the problems and stresses can have a negative impact on MDWs
Hall, Pangan, et al., 2019	NA	*Mental health*: Anxiety	*Social support*: Social network peer support associated with psychological distress
Heng et al., 2019	*Work environment*: Demanding work and daily hours, particularly when caring for older adults *Social isolation*: Long working hours and lack of interaction with co‐workers contributes to social isolation	NA	*Social support*: Employers were identified as the first‐line source of support. Maintaining regular contact with family through messages, phone calls and video calls
Hill et al., 2019	*Workplace abuse*: Vulnerability of MDW to abuse and exploitation in private workplace presents challenges for enforcement of labour law *Work environment*: Demanding work and daily hours	*Physical health*: Increased fatigue postmigration *Mental health*: Stress	NA
Kantaris et al., 2014	*Access to health services*: Unmet needs for health services, pharmaceuticals and low health literacy	NA	*Social support*: Employers were identified as the first‐line source of support
Lo et al., 2019	NA	*Mental health*: Stress	NA
Mendoza et al., 2017	NA	*Mental health*: Increased anxiety, depression, somatization and post‐traumatic stress disorders associated with postmigration stress	*Social support*: Peer sharing of the same problems and stresses can have a negative impact and result in psychological distress
Simkhada et al., 2018	*Workplace abuse*: Physical harm, torture and maltreatment *Access to health services*: Low rates of MDWs receiving health services and poor health literacy associated with health problems *Work environment*: Demanding work and daily hours	*Physical health*: POORER reproductive and sexual health postmigration *Mental health*: Mental health problems	NA
Toyota, 2006	*Workplace abuse*: Sexual abuse by the male employer of male worker *Access to health services*: Low health literature (e.g. poor AIDS knowledge) *Social isolation*: Major stressor. Employers play a major role in constructing social isolation.	*Physical health*: poorer reproductive and sexual health postmigration	NA
Vahabi & Wong, 2017	*Access to health services*: Poor access to healthcare arising from structural exclusion from the healthcare system *Financial hardship*: MDW must endure demanding work conditions as unable to quit domestic work due to financial hardship *Social isolation*: Major stressor	Progressive health declines postmigration *Physical health*: Diabetes postmigration *Mental health*: Anxiety and depression	NA
Van Bortel et al., 2019	*Financial hardship*: Motivation to join the domestic care workforce	Progressive health declines postmigration	*Social support*: Social support not always helpful for coping *Religious activity*: Participating in religious activity coping strategy for stress
van der Ham et al., 2014	NA	NA	*Religious activity*: Praying/reading the bible is a coping strategy for stress (though actual impact not demonstrated)
van der Ham et al., 2015	*Financial hardship*: Income and finances are dominant concerns of MDWs *Work environment*: Demanding work is detrimental to well‐being and does not provide secure long‐term solutions to financial hardship	*Mental health*: Loneliness and homesickness	*Social support*: Employers were identified as first‐line source of support *Religious activity*: Praying/reading the bible is a coping strategy for stress
Wong et al., 2020	NA	NA	Trained Filipina domestic workers showed significantly improved depression literacy, CBT knowledge and attitudes towards seeking professional help. These changes were sustained at a 2‐month follow‐up
Yi et al., 2019	NA	*Mental health*: Mental health issues coupled with poor social support contributed to addictive gambling	NA
Zahreddine et al., 2014	*Work environment*: Demanding work and daily hours	*Mental health*: Moderate to severe depression. Two‐thirds of MDWs diagnosed with a brief psychotic episode	NA

### 2.5 Collating, summarizing and reporting results

In accordance with the guidance provided by Levac et al. (2010), we developed a descriptive summary to collate and summarize the results. Guided by the three‐phase inductive content analysis (Elo & Kyngäs, 2008), the analysis presents a narrative description and synthesis of the existing literature. In the preparation phase, the first and second authors selected the unit of analysis and performed coding using the three research questions as a guiding framework. In the latter organization phase, the third and fourth authors developed the charting tables as analysis matrice. Together with the first author, the third author group data into the charting table to form a category according to the three research questions. The fourth author reviews the content grouped into the charting tables. Finally, the narrative synthesis was reviewed by the fifth, the sixth and the seventh author with reference to the charting table (Elo & Kyngäs, 2008).

### 2.6 Ethics

This study does not need informed consent for patients and ethical approval by an institution.

## 3 RESULTS

The 27 included studies encompassed qualitative (*n* = 8), quantitative (*n* = 14) and mixed methods studies (*n* = 5). Twenty‐one studies focussed specifically on workers from the Philippines (*n* = 14), Indonesia (*n* = 2), Ethiopia (*n* = 2), Nepal (*n* = 1), Burma (*n* = 1) and Russia (*n* = 1). Other studies included a mixture of participants from Philippines, Indonesia, Colombia, Morocco, Senegal, Romania, Sir Lanka, Burma, Vietnam, Sri Lanka, India and Bangladesh. We discuss below the key themes, which comprised stressors impacting health, physical and mental health problems and coping strategies.

### 3.1 Stressors impacting health

Four categories of psychosocial issues, namely: (1) work‐related abuse and exploitation, (2) poor health literacy and accessibility to health services and health insurance, (3) inescapable financial hardship despite a demanding working environment and (4) social isolation, were identified as dominant stressors influencing the health of MDWs.

#### 3.1.1 Work‐related abuse and exploitation

Work‐related abuse and exploitation were popular among MDWs internationally. Work‐related abuses were typically described in categories of sexual abuse, physical abuse, emotional abuse (including verbal abuse) and exploitation, with varying prevalence across countries or cities. The prevalence of abuses experienced by MDWs in Israel was particularly disturbing: sexual abuse (8.2%), physical abuse (5.9%), emotional abuse (38.8%) and exploitation (64.7%) (Green & Ayalon, 2016). Their latter study further showed that MDWs (7.4%) were more vulnerable than local care workers (2.5%) to work‐related abuses. Another survey of Nepalese female MDWs in the Middle East and Malaysia showed that 40.9% suffered abuses in the workplace, including reports of physical harm (11.1%) and torture or maltreatment (30.8%) (Simkhada et al., 2018). In Hong Kong, 20.5% and 34.4% of MDWs experienced physical and verbal abuses, respectively (Cheung et al., 2019). One noteworthy case in Thailand revealed that a male MDW suffered sexual abuses by a male employer repeatedly without reporting to officials or friends (Toyota, 2006). Meanwhile, Green and Ayalon (2018) found that MDWs' worker rights were violated in the following ways: (i) did not receive vacation days (58.4%), did not receive paid sick days (76.1%), did not receive written contracts (15.8%), did not receive the financial compensation they were entitled to (39%) and did not receive weekly days off (35%). In North America, Canadian studies suggested that the household, as a private workplace, presented significant difficulties for officials to enforce existing labour laws, leaving MDWs highly vulnerable to abuses and exploitation with a sense of powerlessness (Hill et al., 2019; Vahabi & Wong, 2017).

#### 3.1.2 Poor health literacy and accessibility to health services and health insurance

Accessibility to health services and health insurance created difficulties for MDWs across both low and middle‐income countries (LMIC) and high‐income countries. Specifically, health illiteracy was identified as a contributing factor. The unmet need for health services (18%), unmet need for pharmaceuticals (10%) and high levels of health illiteracy (73.8%) were reported in one Cyprus quantitative study (Kantaris et al., 2014). Toyota (2006) commented on the impact of health illiteracy on MDW in Thailand, where poor AIDS knowledge left MDWs with extremely restricted options for their own health and well‐being. Simkhada et al. (2018) revealed that only 12.9% of Nepalese MDWs received workplace health services in the Middle East and Malaysia and illiteracy was significantly associated with their health problems (*p* < .001). In Canada, accessibility to health services for MDWs was dependent upon compulsory insurance coverage provided by employers (Carlos & Wilson, 2018). However, there were cases where MDWs lacked awareness about the legal requirement for employers to provide insurance coverage, resulting in no insurance coverage for health services during their first 3 months of employment (Carlos & Wilson, 2018). Systemic inequity, in which MDWs were structurally excluded from healthcare systems of host countries, was found to be a dominant contributor to their poor accessibility in both high‐income (e.g. Canada) and LMIC countries (e.g. Lebanon) (Fernandez, 2018; Vahabi & Wong, 2017).

#### 3.1.3 Inescapable financial hardship despite demanding working environment

Being an MDW is a highly demanding job, yet does not necessarily lift him/her out of poverty. One Singaporean qualitative study revealed that overcoming poverty motivated migrants to join the domestic care workforce in the host countries (Van Bortel et al., 2019). This was confirmed in a Filipino study where financial (52.8%) and income (43.9%) concerns were dominant among MDWs (van der Ham et al., 2015). However, domestic work is highly demanding, and the daily hours of work range from 12 to 18 hr (Hill et al., 2019; Simkhada et al., 2018; Zahreddine et al., 2014). The domestic work was even more demanding if older adults were the care recipients. The daily hours of work increased to 20 hr for MDWs caring for older adults in Singapore (Heng et al., 2019). Particularly, caring for older adults cost an emotional toll on MDWs (Ahonen, et al., 2010). The highly demanding domestic work is detrimental to the well‐being of MDWs and does not bring a permanent solution to MDWs' financial problems because the hardship returns upon termination of employment (van der Ham et al., 2015). This is best explicated by one participant's quote “*I am so stressed and actually I was on sick leave for 12 days because of the stress but what I keep worrying is about my family that if I lose my job right now, because I know I’m not happy with it… If I leave this job and I cannot find a job tomorrow then I lose pay*” (Vahabi & Wong, 2017, p. 8). As such, quitting domestic work is not an option for MDWs, despite enduring (Anbesse et al., 2009).

#### 3.1.4 Social Isolation

MDWs also suffered social isolation as imposed by their employers and being away from their family members. Social isolation was identified as a major issue related to domestic work in various qualitative studies (Ahonen et al., 2010; Anbesse et al., 2009; Carlos & Wilson, 2018; Heng et al., 2019; Toyota, 2006; Vahabi & Wong, 2017). Around 48% of MDWs suffered mild to severe levels of social isolation in Singapore (Anjara et al., 2017). Employers were found to play a major role in constructing the social isolation of MDWs in various ways from forbidding MDWs to connect with the outside world through telephone, to more extreme measures such as fencing them within the home (Toyota, 2006). Meanwhile, the long working hours of domestic care and lack of alternative co‐workers contributed to workplace isolation, in which MDWs carried out their work with no one to talk to or interact with (Ahonen et al., 2010; Heng et al., 2019). Another reported source of social isolation was being separated from family (Carlos & Wilson, 2018), reinforcing their sense of otherness in host countries (Anbesse et al., 2009).

### 3.2 Physical and mental health problems

Both physical and mental health problems were popular across MDWs worldwide with a phenomenon of the healthy immigrant effect. The healthy immigrant effect refers to progressive health declines experienced by MDWs in host countries, after arriving in their host countries with excellent health (Van Bortel et al., 2019). In a Canadian study, MDWs commented that the nature of domestic work (e.g. the live‐in arrangement, demanding work) created new physical and mental health problems that were absent before migration (Carlos & Wilson, 2018).

#### 3.2.1 Physical health

Physical health problem was a common theme across most of the included studies. Various types of physical problems were mentioned across a number of qualitative studies, including cardiovascular diseases (e.g. hypertension) (Carlos & Wilson, 2018; Fernandez, 2018; Hall, Garabiles, & Latkin, 2019; Vahabi & Wong, 2017), respiratory diseases (e.g. tuberculosis) (Fernandez, 2018), chronic pain (Fernandez, 2018; Hall, Garabiles, & Latkin, 2019), diabetes (Hall, Garabiles, & Latkin, 2019; Vahabi & Wong, 2017), poor sleep (Ahonen et al., 2010; Carlos & Wilson, 2018) and fatigue (Ahonen et al., 2010; Hill et al., 2019). Survey data on the above physical problems were absent in the literature that we retrieved. While these physical issues might be attributed to the stressors discussed above (e.g. long working hours) (Carlos & Wilson, 2018), some stressors (e.g. poor sleep and fatigue) were also signs and symptoms of somatization of mental issues (Ahonen et al., 2010; Mendoza et al., 2017). A study in Hong Kong investigated the oral health status of MDWs in which psychosocial determinants explained 13.2% of the variance of caries severity (Gao et al., 2014). Reproductive and sexual health was also a major concern for MDWs (Toyota, 2006). In a study of 1,001 MDWs in Malaysia and the Middle East, 3.1% had fallen pregnant in the course of their employment; 50% of these cases (*n* = 16) was the result of rape/sexual abuse, while the remainder of pregnant women had consensual sexual relationships. Such pregnancies created additional problems for MDWs as sexual relations are forbidden in these Muslim countries (Simkhada et al., 2018). With poor accessibility to health services, there were cases in Lebanon where pregnant MDWs were denied admission to the hospital and were forced to deliver at home (Fernandez, 2018).

#### 3.2.2 Mental health

Mental health issues (e.g. stress, loneliness, anxiety and depression) were commonly reported among MDWs. Feeling stressed is commonly found in MDWs in various studies (Anjara et al., 2017; Bagley et al., 1995; Carlos & Wilson, 2018; Hill et al., 2019; Lo et al., 2019). In Singapore, 52.5% of MDWs reported feeling stress (Anjara et al., 2017). A survey in the Middle East and Malaysia showed that 8.8% of MDWs had mental health problems, although the diseases were not specified (Simkhada et al., 2018). Anxiety and depression were frequently mentioned by MDWs in qualitative studies (Ahonen et al., 2010; Carlos & Wilson, 2018; Hall, Garabiles, & Latkin, 2019; Vahabi & Wong, 2017). The prevalence of anxiety among MDWs was 17.6% in Macao, China (Hall, Pangan, et al., 2019). Ten percent to 18.2% of MDWs suffered moderate to severe levels of depression across various countries (Bagley et al., 1995; Cheung et al., 2019; Hall, Pangan, et al., 2019; Zahreddine et al., 2014). Two studies in Macao and China showed that postmigration stress was associated with increased anxiety, depression, somatization and post‐traumatic stress disorders (Garabiles et al., 2019; Mendoza et al., 2017). The mental health issues, coupled with poor social support quality, contributed to the additive gambling behaviour of MDWs in Macao, China (Hall, Garabiles, & Latkin, 2019; Yi et al., 2019). Loneliness and homesickness were mentioned in MDWs' narratives (Anbesse et al., 2009) with a prevalence of 24.8% in a Filipino study (van der Ham et al., 2015). It is noteworthy that 66.7% of MDWs were diagnosed with brief psychotic episodes in Lebanon (Zahreddine et al., 2014).

### 3.3 Coping strategies

Most reported strategies to cope with stress and to foster resilience were: (1) establishing social networks and (2) participating in religious activities. Particularly, training para‐professional peers was identified as a highly feasible interprofessional intervention to support the mental health of MDWs.

#### 3.3.1 Establishing social networks

Establishing social networks with other MDWs through various means is a popular strategy for MDWs to seek help. Yet, it might not be entirely beneficial for MDWs. For Ethiopian MDWs, establishing social networks and contact with peers served the function of sharing information and advice, engaging in religious worship together and comforting themselves just by seeing each other (Anbesse et al., 2009). Those MDWs with higher levels of social connection expressed higher levels of quality of life in Singapore (Anjara et al., 2017). However, the social network is not always beneficial. For MDWs in Macao, China, social support by peers, as mutual aids, was positively associated with psychological distresses (Hall, Pangan, et al., 2019; Mendoza et al., 2017). It was suggested that peers shared the same problems and were similarly stressed (Hall, Garabiles, & Latkin, 2019). Some MDWs in Singapore indicated that social support from peers was not helpful (Van Bortel et al., 2019). On the other hand, employers were identified as a prominent and first‐line source of support (inclusive instrumental, emotional and financial) for MDWs in various studies (Heng et al., 2019; Kantaris et al., 2014; van der Ham et al., 2015). However, among those being abused, less than 50% of MDWs reported abuse by employers, either formally to officials or informally to friends and family (Green & Ayalon, 2016). Motivating them to persevere, MDWs also tried to maintain regular contact with their family members through messages, phone calls and video calls (Heng et al., 2019). In some settings, there were cases where social contact with friends and family was prohibited by employers of MDWs (Anbesse et al., 2009). A study in Singapore focussed on training 40 Filipino domestic workers to be para‐professional peer counsellors with basic cognitive behavioural therapy (CBT). Encouragingly, trained Filipino domestic workers demonstrated significant improvement in depression literacy (Wong et al., 2020), CBT knowledge and attitudes towards seeking professional help. The training was also well accepted by the participants (Wong et al., 2020).

#### 3.3.2 Participating in religious activities

Religion was a very important aspect of the resilience of many MDWs and religious activities performed included singing, prayer, going to church and reading religious texts (Van Bortel et al., 2019). In the Philippines, praying/reading the Bible (55.2%) was a major strategy for MDWs to cope with stress (van der Ham et al., 2015), although the stress levels between those who prayed/read the Bible and those who did not show no differences (van der Ham et al., 2014).

## 4 DISCUSSION

This is the first scoping review and synthesis of literature related to the health issues of MDWs involving 16 countries or cities globally. It indicates that MDWs' health is a global issue and provides a valuable addition to the literature on the health inequity of MDWs. From a human rights perspective, identifying stressors and subsequent coping strategies used by MDWs to address their health problems is merited. Human rights issues are meant in the first instance to guide the actions of governments and have implications for health policy and practice (Gruskin et al., 2007). One systematic review identified that common stressors, which triggered emotional and psychological problems in MDWs included, abusive behaviour by employers, sexual harassment, economic exploitation, demanding working environment and recurrent financial hardships (Wang & Wu, 2017), all consistent with the findings of our review.

The “healthy immigrant effect” was originally employed to permanent migrants in migrant studies (Anikeeva et al., 2010). For example, a review of permanent migrants in Australia showed that the migrant health advantage generally deteriorates with increasing duration of residence (Anikeeva et al., 2010). A similar situation of the general deterioration of health stats was also experienced by MDWs in our findings. Indeed, the “healthy immigrant effect” was shown to be experienced by temporary foreign workers as well (Vahabi & Wong, 2017). This scoping review identified a range of physical problems, such as chronic pain, fatigue, sleeping difficulties, gambling behaviour and suicide, indicating the potential of somatization of mental problems faced by MDWs. High levels of stress, anxiety and depressive symptoms were common in MDWs across all geographical regions. However, these problems were largely reported in qualitative studies, in which systematic survey data on physical and mental problems, inclusive sexual and reproductive health, faced by MDWs are lacking in this scoping review. A recent survey using summary scores on physical health and mental health showed that MDWs suffered poorer physical and mental health status when compared with the general population in Hong Kong (Chung & Mak, 2020), further providing solid evidence on the health inequalities of MDWs. However, the types of physical and mental problems faced by MDWs were not identified by Chung and Mak (2020). As such, our findings highlight the importance for health and social care researchers to systemically assess the physical and mental problems of MDWs, actively listen to their concerns and demonstrate commitment to support and follow‐up.

In coping with stressors and health problems, numerous socio‐cultural disadvantages (e.g. social isolation, marginalization from health and social care systems, language barriers) contributed to their poor accessibility to health services and limited health literacy in host countries (Weng et al., 2021). As such, they faced challenges in accessing and understanding health‐related information and services (Ho & Smith, 2020). In our findings, MDWs identified employers as a major source of social support (Heng et al., 2019; Kantaris et al., 2014; van der Ham et al., 2015). However, our findings also showed that employers were a possible source of abuse for MDWs. It was shown that employers could be a source of stress because of the unequal employer‐employee power relationship (Ho et al., 2019). While the live‐in arrangement prevented law enforcement in the private sphere to protect MDWs, MDWs are at the same time dependent on their employers for instrumental support, informational support and emotional support (Ho et al., 2019). This complex situation exacerbates the vulnerabilities of MDWs, in which nurses in the community need to partner with social care professionals in order to address health issues within a marginalized social environment.

As an advocate of clients, providing health literacy support to clients is a core nursing skill (Wittenberg et al., 2018). Our findings on peer support and participating in religious activities shed light on the possible way for nurses in community settings to mitigate the health inequity of MDWs, particularly addressing the issues of structural exclusion from healthcare system and limited health literacy. MDWs mainly shared information and advice by establishing social networks and contacts with peers (Anbesse et al., 2009). However, providing or receiving mutual aids among peers was not always entirely positive because they shared similar problems with limited resources (Hall, Garabiles, & Latkin, 2019). Building on MDWs' preference to seek help from peers, in which mutual aids may not be always effective, training peer leaders to be para‐professional peer counsellors was shown to be a feasible and potentially effective intervention to promote the mental well‐being and mental health literacy of MDWs (Wong et al., 2020). Wong et al. (2020) showed that peer support coupled with training of CBT was effective to improve the mental health literacy of MDWs, and a potential bridge between formal healthcare services and MDWs. However, the study of Wong et al. (2020) was a pilot study with 37 participants. Meanwhile, it was commented that CBT might be too intensive for MDWs, as such mental health first aid training could be an alternative (Ho et al., 2022; Hung et al., 2021). Further large‐scale randomized controlled trial is needed for strong empirical evidence of the effectiveness of para‐professional training of MDWs to improve their mental health.

According to our findings, MDWs were marginalized from health and social care services. This will certainly present a challenge to nurses in secondary prevention settings to identify MDWs in distress. A recent study has shown potential in training up peer support workers and including them in interprofessional teams for more egalitarian service provision for people with mental health issues (Ehrlich et al., 2019). Given nurses bears the role of coordinator, this may be particularly helpful to actively involve marginalized MDWs. On the other hand, our findings have also shown that MDWs performed religious activities in church. As such, collaborating with faith‐based organizations is a possible way for nurses in the community to collaborate with social care professionals to provide peer support programmes for MDWs in the community. As an example, a community engagement forum in Hong Kong led by nurses was shown to be feasible to engage the MDW community to co‐create peer support with the non‐government organization (The Nethersole School of Nursing, The Chinese University of Hong Kong, 2022).

Small sample size and non‐probability sampling were common in quantitative studies, highlighting a research gap in current literature to produce a more rigorous design to ensure better generalization. Several mixed methods research studies included in this review were designed to capture experiential aspects of the health‐related consequences of working as an MDW. We further advocate for a participatory action research approach to actively empower MDWs through the co‐construction of a peer support programme. Bhuyan et al. (2018) demonstrated an excellent example of advocacy for migration policy changes for MDWs in Canada, using a participatory action research approach. The changes were deemed to be important to ameliorate the structural violence of migrant labour (Bhuyan et al., 2018).

### 4.1 Limitations

There are limitations in this scoping review. Firstly, the inductive nature of scoping review with the diversity of countries included in this review risks the potential for lack of specificity. Secondly, scoping reviews are designed to map the evidence and generally do not exclude studies on the grounds of quality appraisal; hence, caution should be paid when interpreting findings from studies with poor quality. Thirdly, this review only included English language literature because screening of Chinese and Indonesian papers did not identify any eligible articles. Studies in other languages (e.g. Filipino) might provide more contextualized findings for the readers. Fourth, the COVID‐19 pandemic may have exacerbated employment constraints and had a negative effect on MDWs' experiences. However, this review was unable to provide information on the impact of the COVID‐19 pandemic on this group.

## 5 CONCLUSIONS

Narrowing health inequity has long been the heart of helping professions. As advocators, nurses have the responsibility to improve the accessibility of MDWs to healthcare services and their health literacy. Our review findings illustrate MDWs as a vulnerable group with extreme health inequity, for whom marginalization requires nurses to reach out to the community and to include them in interprofessional teams. Taking approaches to train para‐professional peer leaders with CBT can be a possible way to support the health and well‐being of MDWs. To reach migrant domestic workers who are systematically marginalized from health and social care services, engaging faith‐based organizations could be potentially effective. Employing a participatory action approach will be a feasible way to co‐create a suitable peer support programme for MDWs.

## AUTHOR CONTRIBUTIONS

Ken, Ingrid, Janet and Graeme drafted the manuscript. Sonia, Lisa and Ferry reviewed and revised the manuscript. All authors have reviewed the submitted manuscript and approved the manuscript for submission. All the team members participated in the screening of articles.

## CONFLICT OF INTEREST

We declare no conflict of interestPreprint version.

## APPENDIX 1

### Ovid medline

**Table:**

#	Searches
1	exp migrant worker/
2	(domestic helper or domestic worker or foreign worker or domestic employee).ti,ab,hw
3	or/1‐2
4	exp health
5	exp well‐being
6	exp psych*
7	exp physical
8	exp work like
9	(mental or emotional). ti,ab,hw.
10	or/4‐10
11	4 and 10
12	Limit 11 to (humans and yr="1995 ‐ 2019")
